# Human Rho Guanine Nucleotide Exchange Factor 11 (ARHGEF11) Regulates Dendritic Morphogenesis

**DOI:** 10.3390/ijms18010067

**Published:** 2016-12-29

**Authors:** Yutaka Mizuki, Manabu Takaki, Shinji Sakamoto, Sojiro Okamoto, Makiko Kishimoto, Yuko Okahisa, Masahiko Itoh, Norihito Yamada

**Affiliations:** 1 Department of Neuropsychiatry, Okayama University Graduate School of Medicine, Dentistry and Pharmaceutical Sciences, 2-5-1 Shikata-cho, Kita-ku, Okayama 700-8558, Japan; yutaka0321@gmail.com (Y.M.); shinjisakamoto1202@gmail.com (S.S.); sou060646@gmail.com (S.O.); kiko973@hb.tp1.jp (M.K.); okahis-y@cc.okayama-u.ac.jp (Y.O.); nyamada@okayama-u.ac.jp (N.Y.); 2 Department of Biochemistry, Dokkyo Medical University School of Medicine, 880 Kitakobayashi, Mibu-machi, Shimotsuga-gun, Tochigi 321-0293, Japan; mitoh@dokkyomed.ac.jp

**Keywords:** ARHGEF11, schizophrenia, PSD-95, synaptophysin, immunoprecipitation, dendritic spine

## Abstract

Disturbances of synaptic connectivity during perinatal and adolescent periods have been hypothesized to be related to the pathophysiology of schizophrenia. Rho guanine nucleotide exchange factor 11 (ARHGEF11) is a specific guanine nucleotide exchange factors (GEF) for RhoA, which is a critical regulator of actin cytoskeleton dynamics and organization of dendritic spines and inhibitor of spine maintenance. *ARHGEF11* variants are reported to be associated with a higher risk for the onset of schizophrenia in a Japanese population; however, how ARHGEF11 contributes to the pathogenesis of schizophrenia in dendritic spines is unknown. Therefore, we first studied the distribution, binding, and function of ARHGEF11 in the dendritic spines of the rat cerebral cortex. After subcellular fractionation of the rat cerebral cortex, ARHGEF11 was detected with synaptophysin and post-synaptic density protein 95 (PSD-95) in the P2 fractions including synaptosomal fractions containing presynaptic and postsynaptic density proteins. Endogenous ARHGEF11 was coimmunoprecipitated with synaptophysin or PSD-95. In cortical primary neurons at 28 days in vitro, immunostaining revealed that ARHGEF11 located in the dendrites and dendritic spines and colocalized with PSD-95 and synaptophysin. Overexpression of exogenous ARHGEF11 significantly decreased the number of spines (*p* = 0.008). These results indicate that ARHGEF11 is likely to be associated with synaptic membranes and regulation of spine.

## 1. Introduction

Disturbances in synaptic connectivity during perinatal and adolescent periods underlie the pathophysiology of schizophrenia [1]. Postmortem brain studies of individuals with schizophrenia have reported reduced dendritic spine density in the cerebral neocortex [2,3,4,5]. These dendritic spine abnormalities are likely the result of disturbances in the molecular mechanisms that contribute to spine formation, pruning, and/or maintenance [6]. Candidate genetic factors for schizophrenia, including disrupted in schizophrenia 1 (DISC1), neuregulin-1 (NRG1), and dysbindin, have distinct roles in synapse-specific mechanisms and directly affect functional signaling involved in synaptic transmission [7].

Dendritic spines are tiny dendritic protrusions that harbor the majority of excitatory synapses in the central nervous system (CNS) [8] and exhibit structural modification in response to synaptic activity [9,10]. Dendritic spine morphogenesis is regulated through cytoskeletal actin, which is highly concentrated in the spines [11,12]. The Rho family of small GTPases (Rho GTPases), which includes Cdc42, Rac1, and RhoA, is a critical regulator of actin cytoskeleton dynamics and organization in the spines [13,14]. A variety of evidence indicates that Rac1 promotes spine formation, while RhoA inhibits spine maintenance [15,16]. The activation of Rho GTPases is mediated by specific guanine-nucleotide exchange factors (GEFs) that catalyze the exchange of bound guanosine diphosphate (GDP) (inactive state) for bound guanosine triphosphate (GTP) (active state) [17]. Several Rho GEFs that localize to dendritic spines play important roles in dendritic spine morphogenesis by modulating the activity of Rho GTPases [18,19]. Of these Rho GEFs, it is reported that kalirin-7 is enriched in dendritic spines through interaction with post-synaptic density 95 (PSD-95), a major scaffolding protein in the post-synaptic density of the glutamate synapse [20]. Kalirin-7 also interacts with genetic factors for schizophrenia (e.g., DISC1, NRG1) in regulating dendritic morphology and spine plasticity [21,22].

Rho guanine nucleotide exchange factor 11 (ARHGEF11), which is also called PDZ-RhoGEF and KIAA0380, is a specific GEF for RhoA, and does not interact with Rac1 or Cdc42 [23]. ARHGEF11 is highly expressed in the brain [24,25] and involved in actin cytoskeletal reorganization [26]. The rat homolog of ARHGEF11, GTRAP48, has been shown to directly bind with excitatory amino acid transporter 4 (EAAT4). This interaction is important for the glutamate uptake activity of EAAT4 [25]. We found that *ARHGEF11* variants are associated with a higher risk for the onset of schizophrenia in a Japanese population [27]. Thus, altered *ARHGEF11* expression may play a role in the pathophysiology of schizophrenia.

The way ARHGEF11 in dendritic spines contributes to the pathogenesis of schizophrenia is unknown, thus we studied the distribution, binding, and functions of ARHGEF11 in the dendritic spine of the rat cerebral cortex.

## 2. Results

### 2.1. Subcellular Distribution and Localization of ARHGEF11 in Rat Cerebral Cortex

**Figure 1:**
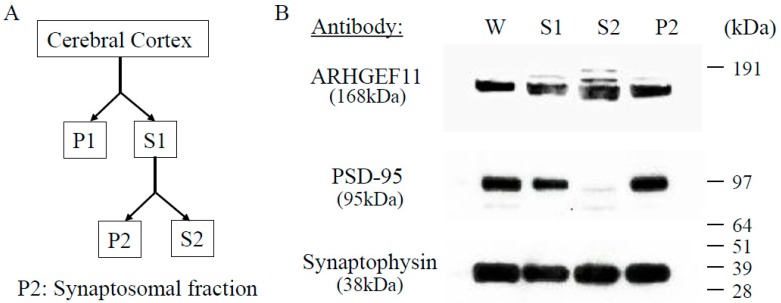
Subcellular distribution and localization of ARHGEF11 in rat cerebral cortex: the method of fractionating cerebral cortex (**A**); and fractions with antibodies against ARHGEF11, synaptophysin, and PSD-95 (**B**). ARHGEF11, synaptophysin, and PSD-95 were detected in the crude synaptosomal fractions (P2); S1 (supernate 1), S2 (supernate 2). P1 (pellet 1).

To characterize the subcellular localization of ARHGEF11, we fractionated homogenates of rat cerebral cortex and analyzed the fractions with antibodies directed against ARHGEF11, synaptophysin, and post-synaptic density protein 95 (PSD-95) (Figure 1B). As expected, synaptophysin and PSD-95 were enriched in the crude synaptosomal fractions containing pre- and postsynaptic density proteins (P2). ARHGEF11 immunoreactivity was also detected in the P2 fractions. These results indicate that ARHGEF11 is likely to be associated with synaptic membranes and activity.

### 2.2. Complex Formation of ARHGEF11 and Synaptic Marker Proteins

**Figure 2:**
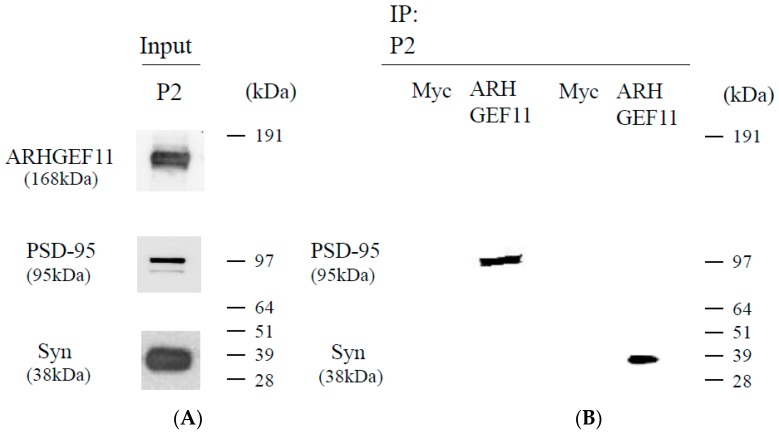
Formation of ARHGEF11 and synaptic marker protein complex. The immunoprecipitation of synaptophysin and PSD-95 with ARHGEF11 (Acris) or negative control (Myc antibody): input (**A**); and immunoprecipitation (**B**). ARHGEF11 was coimmunoprecipitated with synaptophysin (38 kDa) and PSD-95 (95 kDa) in P2 fractions (**B**). Three independent experiments were conducted.

Since ARHGEF11 is concentrated in P2 fractions (Figure 1), we examined its binding to two synaptic proteins: synaptophysin (presynaptic) and PSD-95 (postsynaptic). ARHGEF11 was coimmunoprecipitated with synaptophysin and PSD-95. Interactions of ARHGEF11/synaptophysin and ARHGEF11/PSD-95 were observed in the P2 fraction (Figure 2).

### 2.3. Localization of Endogenous ARHGEF11 in Cortical Primary Neurons

**Figure 3:**
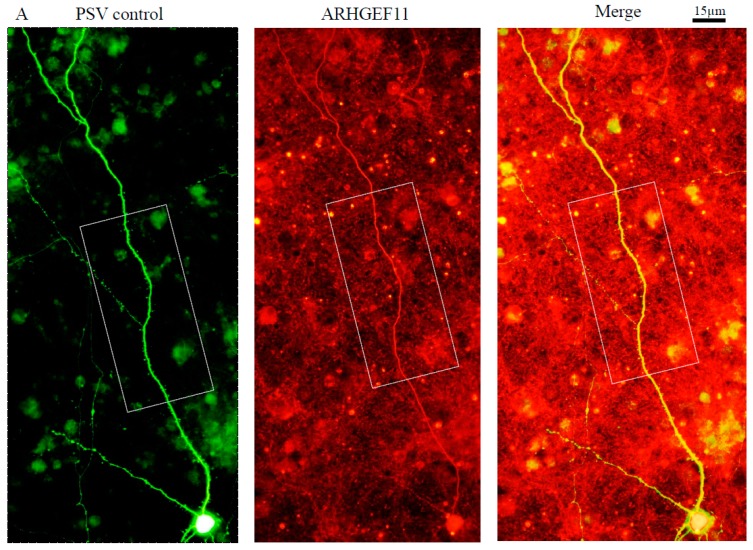

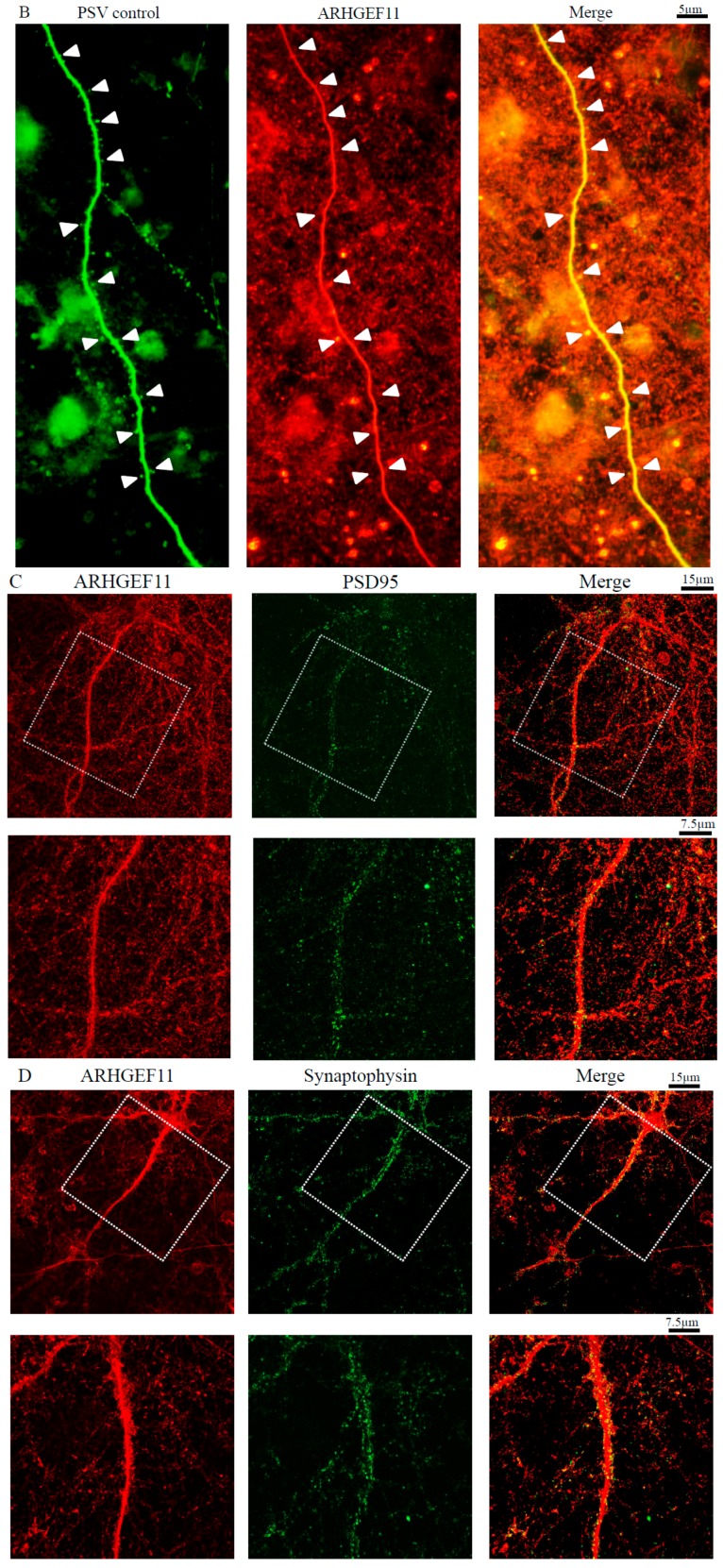
Localization of endogenous ARHGEF11 in cortical primary neurons. Immunofluorescent cell staining was conducted. Expression of ARHGEF11 in primary cortical neurons at 28 day in vitro (D.I.V.): ARHGEF11 (red) located in the dendrite and dendritic spine (**A**,**B**); ARHGEF11 (red) was colocalized with PSD-95 (green) at the punctate structures of dendrites (**C**); and ARHGEF11 (red) was colocalized with synaptophysin (green) (**D**). Three independent experiments were conducted. Dotted rectangles indicate the area of lower figure. White arrows indicate merged spines.

To determine the subcellular localization of ARHGEF11 in the cortical primary neurons, we investigated the expression of ARHGEF11 in primary cortical neurons at 28 day in vitro (D.I.V.), which had mature synapses with fully differentiated postsynaptic densities. Immunostained images revealed that ARHGEF11 was located in the dendrites and dendritic spines (Figure 3A,B). Furthermore, it was found that ARHGEF11 was colocalized with PSD-95 at the punctate structure of dendrites, suggesting the localization of ARHGEF11 to dendritic spines (Figure 3C). ARHGEF11 was also colocalized with synaptophysin (Figure 3D).

### 2.4. Regulation of Spine Formation by ARHGEF11

**Figure 4:**
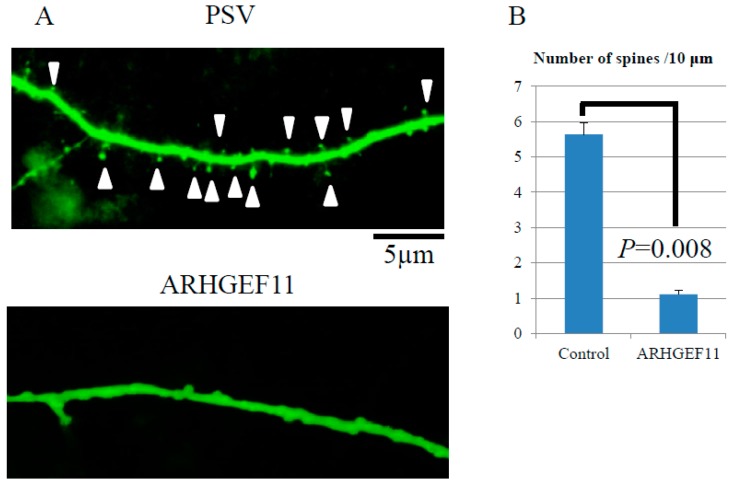
Regulation of spine formation by ARHGEF11. pSuper Venus (green) and Exo-ARHGEF11 construct were transfected in cortical neuron at 26 days in vitro. After the immunofluorescent cell staining at 28 days, the number of dendritic spines was analyzed over 10,000 µm of dendritic tissue from eight independent experiments using Lumina Vision. Overexpression of Exo-ARHGEF11 significantly decreased the number of spines (*p* = 0.008) (**A**,**B**). White arrows indicate spines.

Finally, to investigate whether ARHGAEF11 regulates spine formation, ARHGEF11 was overexpressed in primary cultured neurons by transfection with Lipofectamine. Overexpression of Exo-ARHGEF11 significantly decreased the number of spines (*p* = 0.008) (Figure 4A,B).

## 3. Discussion

In this study, we demonstrated that ARHGEF11 interacts and colocalizes with synaptophysin and PSD-95 at synapse sites. Furthermore, ARHGEF11 negatively regulated the formation of dendritic spines in cortical primary neurons.

Previous studies have shown that candidate genetic susceptibility factors for schizophrenia, DISC1 and dysbindin-1, function both pre- and postsynaptically through interaction with partners to regulate neurotransmitter release and signal transduction [22,28,29]. ARHGEF11 interaction with synaptophysin (presynaptic) and PSD-95 (postsynaptic) in the crude synaptosomal fractions implies that ARHGEF11 also functions both pre- and post-synaptically.

The ARHGEF11 binding partner synaptophysin was the first integral membrane protein of synaptic vesicles (SVs) to be cloned and is expressed in all neurons and endocrine cells [30,31]. Synaptophysin is involved in important aspects of neurotransmitter release via SV exocytosis [32] and recycling via SV endocytosis by functionally interacting with nerve terminal proteins (e.g., synaptobrevin 2 and dynamin) [33,34]. In presynaptic terminals, Rho kinase, a major downstream effector of RhoA accelerates SV endocytosis [35]. Conversely, GEF-H1, another GEF for RhoA, interacts with the exocyst component and activates RhoA, which in turn regulates the vesicle-trafficking pathways of exocytosis [36]. Similarly, ARHGEF11/synaptophysin interaction might affect the formation and trafficking of SVs by regulating the RhoA signal cascade.

On the other hand, the ARHGEF11 binding partner PSD-95 is one of the most abundant scaffolding proteins in PSD proteins [37]. NMDA receptors, a family of ionotropic glutamate receptors at postsynaptic sites, are anchored in PSD proteins by interactions between the cytoplasmic C-terminal domain of their NR2 subunits and the PDZ domains of PSD-95 [38,39]. The PDZ domains of PSD-95 also bind to other postsynaptic membrane proteins including kalirin [20,40]. Such interactions are proposed to be important for localization and clustering of these proteins or organizing signal transduction pathways at the postsynaptic membrane. These signaling complexes are known to play prominent roles in synaptic plasticity [40]. Furthermore, GTRAP48, the rat homolog of ARHGEF11, interacts with the neuronal glutamate transporter EAAT4 and modulates its glutamate transport activity [25]. EAAT4 is highly expressed in the cerebellum, where it is localized to postsynaptic sites of Purkinje cell dendrites and spines, and low-level expression is also observed in the cerebral cortex [41,42,43]. We found ARHGEF11/PSD-95 interaction at synapse sites, and overexpression studies revealed important roles for ARHGEF11 in dendritic formation. Although, the importance of ARHGEF11/PSD-95 interaction is not clear in this study, ARHGEF11 (schizophrenia-related gene *ARHGEF11*) might play a role in glutamate receptor-mediated synaptic plasticity through regulation of RhoA signaling to the actin cytoskeleton.

In a yeast two-hybrid screen, ARHGEF11 interacts with DISC1 [44]. DISC1 directly interacts with PSD-95 and kalirin-7, a GEF for Rac1, and blocks access of kalirin-7 to Rac1. This binding is released by NMDA receptor activation, allowing free access of kalirin-7 to Rac1, and leading to the resultant activation of Rac1 and spine enlargement [22]. In this context, DISC1 might also regulate the access of ARHGEF11 to RhoA, resulting in spine shrinkage. Thus, this regulation of access of GEFs to RhoGTPases by DISC1 might be crucial for the proper maintenance of the dendritic spine.

Dysfunction of insulin release from pancreatic islet β-cells is considered to be one of the causal factors in the etiology of type 2 diabetes mellitus (T2DM). It was discovered that Rac1 was particularly important for glucose-stimulated insulin secretion [45]. In contrast, RhoA expression is increased in β-cells under diabetic conditions, and Rho/Rho-kinase activation is involved in the suppression of insulin biosynthesis [46]. Thus, insulin release from pancreatic islet β-cells could be determined by the resulting balance between RhoA and Rac1 activities. The co-occurrence of schizophrenia and T2DM has been well documented. Schizophrenia and T2DM are common diseases that seem to share a complex mode of inheritance that includes both genetic factors and environmental determinants [47,48]. It is reported that *ARHGEF11* variants are associated with schizophrenia [27] and T2DM in multiple ethnic populations [49,50,51,52,53]. Taken together, these findings suggest that Rho GTPase signaling affects not only the dendritic spine structure, but also insulin release from pancreatic islet β-cells, and that aberrations in Rho GTPase signaling, including its activation by GEFs, could therefore contribute to the comorbidity of schizophrenia and T2DM.

This study has some limitations. We only showed the interaction and colocalization of ARHGEF11 with the presynaptic marker synatptophysin and the postsynaptic marker PSD-95 and dendritic spine morphology by overexpression of ARHGEF11. In future study, we should investigate the suppression of ARHGEF11 by RNAi, the underlying molecular mechanisms of ARHGEF11-mediated spine formation/synaptic plasticity, and whether and how DISC1 interacts with it to control ARHGEF11 localization.

## 4. Materials and Methods

### 4.1. Antibodies

Antibodies to ARHGEF11 (Acris, Herford, Deutschland), PSD-95 (Affinity BioReagents, Golden, CO, USA), synaptophysin (Sigma-Aldrich, St. Louis, MO, USA), and a conjugated anti-mouse or anti-rabbit secondary antibody (Amersham Pharmacia, Piscataway, NJ, USA) were used for immunostaining, immunoblotting and immunoprecipitation.

### 4.2. Tissue Preparation and Subcellular Fractionization

All procedures involving animals were approved by the Committee for Recombinant DNA Experiments (#11092, 10/09/2013) and the Animal Care and Use Committee (#OKU-2014002, 01/04/2014) of Okayama University Graduate School of Medicine, Dentistry and Pharmaceutical Sciences. The brain was removed from an adult rat after decapitation, and the cerebral cortex was dissected from the other parts. The method of fractionating the cerebral cortex was based on the original methods of Carlin [54]. The procedure is outlined in Figure 1A. In detail, the cerebral cortex was homogenized in 0.32 M ice-cold buffer A containing sucrose (1 M sucrose, 1 mM NaHCO_3_, 1 mM MgCl_2_, 0.5 mM CaCl_2_). Tissues were homogenized by 12 up and down strokes with a Teflon-glass homogenizer, using 1 g (wet weight) cortex/4 mL of solution A. The first fraction was centrifuged (1400× *g*; *g* values are average centrifugal forces) for 10 min, and a low-speed pellet was obtained. Next, the second centrifugation (710× *g*) of the supernate was carried out for 10 min. After the second centrifugation, the supernate (S1) was centrifuged at 13,800× *g* for 10 min. The supernate (S2) and the resulting crude synaptosomal pellet containing pre-synaptic and post-synaptic density proteins (P2) were obtained.

### 4.3. Western Blotting

Each tissue (cerebral cortex, supernate S1, supernate S2, and crude synaptosomal pellet P2) was mixed with a NuPAGE SDS-PAGE loading buffer (Thermo Fisher Scientific, Waltham, MA, USA) containing β-mercaptoethanol immediately after measurement of protein concentrations. The first antibodies were ARHGEF11 (Acris, 1:300), post-synaptic density protein 95 (PSD-95) (Affinity BioReagents, 1:500), and synaptophysin (Sigma-Aldrich, 1:500). A secondary antibody for Western blotting was anti-mouse or -rabbit IgG conjugated with horseradish peroxidase (GE Healthcare Bioscience, Buckinghamshire, UK). For detection, we used an enhanced chemiluminescence reagent, ECL Plus Western Blotting Detection System (GE Healthcare Bioscience, Buckinghamshire, UK).

### 4.4. Immunoprecipitation

The tissue (crude synaptosomal pellet P2) was lysed in lysis buffer (150 nM NaCl, 50 mM Tris-HCl (pH 7.6), 1% NP-40, and a protein inhibitor cocktail (cOmplete, Mini, EDTA-free, Roche Life Sciences, Indianapolis, IL, USA)). Supernatant fractions obtained after centrifugation at 10,000× *g* for 20 min were incubated with 5 µL of the ARHGEF11 (Acris) specific polyclonal antibodies overnight, which was followed by the addition of 30 µL protein A agarose (Thermo Fisher Scientific). The immunoprecipitates were analyzed by NuPAGE SDS-PAGE (Thermo Fisher Scientific), followed by Western blotting. An antibody to c-Myc (9E10) (Santa Cruz Biotechnology, Santa Cruz, CA, USA) was used as a negative control for immunoprecipitation.

### 4.5. Neuron Culture and Treatment

Dissociated cortical neurons from Sprague-Dawley rats at 18 days gestation (E18) were plated at a density of 3 × 10^5^ cells/mL on precoated coverglasses (Matsunami, Osaka, Japan) and cultured in a humidified atmosphere of 5% CO_2_ at 37 °C for 28 days. One half of the medium was exchanged twice every week. We followed the “Fundamental Guidelines for Proper Conduct of Animal Experiment and Related Activities in Academic Research Institutions”.

### 4.6. Plasmid Construction and Transfection

Myc-ARHGEF11 was a gift from Masahiko Itoh (Dokkyo Medical University, Tochigi, Japan) [55]. Cortical neurons were transfected with plasmids using Lipofectamine 2000 (Thermo Fisher Scientific). In primary neuron cultures, we transfected 3 µg of pSuper Venus construct into 3.0 × 10^5^ cells. In standard analyses, primary neurons were transfected after 26 D.I.V. and maintained for 1–2 day(s) after transfection.

### 4.7. Immunofluorescent Cell Staining

Experimental procedures for cell staining were described previously [56]. In brief, cells were fixed with 3.7% paraformaldehyde in phosphate-buffered saline (PBS) and permeabilized with 0.1% Triton X-100. After blocking with 1% donkey serum albumin (DSA) in PBS, primary antibodies were incubated at 4 °C overnight, and the cells were incubated with a Rhodamine Red-X- or Fluorescein (FITC)-conjugated secondary antibody (Jackson ImmunoResearch, West Grove, PA, USA) at 1:1500 dilution at room temperature for 45 min. Hoechst 33258 (Thermo Fisher Scientific) was used at 1:3000 dilution for 2 min. Confocal microscopy used a Zeiss LSM 510 (Oberkochen, Deutschland) to obtain phase contrast images. For some staining, methanol at −20 °C was used for 10 min as the fixative. An Olympus BX53 microscope, a charge-coupled device (CCD) camera, FX630 (Olympus, Shinjuku, Japan) was used to analyze immunofluorescent staining of transfected cortical neurons.

### 4.8. Statistical Analysis

At 28 D.I.V., the number of dendritic spines was analyzed over 10,000 µm of dendritic tissue from eight independent experiments by Lumina Vision (Mitani Corporation, Fukui and Tokyo, Japan). Statistical analysis was performed by F-TEST (Microsoft Excel, Redmond, WA, USA).

## 5. Conclusions

ARHGEF11 is likely to be associated with synaptic membranes and regulation of spine. Alterations in *ARHGEF11* gene and ARHGEF11 protein expression might contribute to the pathophysiology of schizophrenia.
